# An approach for evaluating the bioavailability and risk assessment of potentially toxic elements using edible and inedible plants—the Remance (Panama) mining area as a model

**DOI:** 10.1007/s10653-021-01086-8

**Published:** 2021-10-22

**Authors:** Ana Cristina González-Valoys, José Ulises Jiménez Salgado, Rita Rodríguez, Tisla Monteza-Destro, Miguel Vargas-Lombardo, Eva María García-Noguero, José María Esbrí, Raimundo Jiménez-Ballesta, Francisco Jesús García-Navarro, Pablo Higueras

**Affiliations:** 1 Centro Experimental de Ingeniería, Technological University of Panama, Vía Tocumen, 0819-07289 Panama City, Panama; 2 Instituto de Geología Aplicada, Castilla-La Mancha University, EIMI Almadén. Plaza Manuel Meca 1, Almadén, 13400 Ciudad Real, Spain; 3 Department of Geology & Geochemistry, Autonomous University of Madrid, University City of Cantoblanco, 28049 Madrid, Spain; 4 Centro de Investigaciones Hidráulicas e Hidrotécnicas, Technological University of Panama, Ricardo J. Alfaro Avenue, Dr. Víctor Levi Sasso University Campus, 0819-07289 Panama City, Panama; 5 Dirección de Investigación, Vicerrectoría de Investigación, Postgrado y Extensión, Technological University of Panama, Ricardo J. Alfaro Avenue, Dr. Víctor Levi Sasso University Campus, 0819-07289 Panama City, Panama; 6 Departamento de Geotecnia, Facultad de Ingeniería Civil, Technological University of Panama, Ricardo J. Alfaro Avenue, Dr. Víctor Levi Sasso University Campus, 0819-07289 Panama City, Panama; 7 Facultad de Ingeniería de Sistemas Computacionales, Technological University of Panama, Ricardo J. Alfaro Avenue, Dr. Víctor Levi Sasso University Campus, 0819-07289 Panama City, Panama; 8 SNI-SENACYT Sistema Nacional de Investigación-Secretaria Nacional de Ciencia, Tecnología e Innovación, Clayton, Ciudad del Saber Edif.205, 0816-02852 Panama City, Panama; 9 Escuela Técnica Superior de Ingenieros Agrónomos de Ciudad Real, Castilla-La Mancha University, Ronda de Calatrava n° 7, 13071 Ciudad Real, Spain

**Keywords:** Potentially toxic elements (PTEs), Plants, Bioavailability, Risk assessment, Food

## Abstract

Mining affects the environment, particularly through the persistence of accumulation of tailings materials; this is aggravated under tropical climatic conditions, which favours the release of potentially toxic elements (PTEs) bioavailable to the local flora and fauna and supposing a risk to human health. The Remance gold mine (Panamá), exploited intermittently for more than 100 years, and has remained derelict for over 20 years. Within the area live farmers who carry out subsistence agriculture and livestock activities. The objective of this study has been to study the transference of PTEs in the local agricultural soil-plants system, with the goal of identifying their bioavailability to perform a human risk assessment. The results obtained of the Bioaccumulation coefficient in local plants show very weak to strong absorption of As (< 0.001–1.50), Hg (< 0.001–2.38), Sb (0.01–7.83), Cu (0.02–2.89), and Zn (0.06–5.32). In the case of Cu in grass (18.3 mg kg^−1^) and plants (16.9 mg kg^−1^) the concentrations exceed the maximum authorised value in animal nutrition for ruminants (10 mg kg^−1^). The risk to human health for edible plants exceeds the non-carcinogenic risk for rice, corn, cassava, and tea leaves for Sb (HQ 19.450, 18.304, 6.075, 1.830, respectively), the carcinogenic risk for Cu (CR = 2.3 × 10^–3^, 7.7 × 10 ^−4^, 1.1 × 10^–3^, 1.0 × 10^–3^, respectively), and the carcinogenic risk for As in rice, corn and tea leaves (CR = 8 × 10^–5^, 3 × 10^–5^, 3 × 10^–5^, respectively). Urgent measures are needed to alleviate these effects.

**Supplementary Information:**

The online version contains supplementary material available at 10.1007/s10653-021-01086-8.

## Introduction

Soil quality is affected by the presence of PTEs, which is largely due to anthropogenic activity (Bravo et al., 2017; Hooda, 2010; Rogival et al., 2007; Zhuang et al., 2009). Mining activity strongly impacts the environment because it implies exposing the minerals that contain PTEs to atmospheric conditions (Kamunda et al., 2016; Palansooriya et al., 2020). In particular, abandoned mining tailings become sources of environmental contamination (Chaabani et al., 2017; Kaninga et al., 2020; Santos et al., 2016) when they are exposed to environmental conditions like rain and wind, which influences the entire food chain from soils to plants and animals and, directly or indirectly, to human beings (Getaneh & Alemayehu, 2006).

For example, Cu is an essential micronutrient, which participates in the transfer of electrons, but it can be toxic to plants and humans in large quantities (Bravo et al., 2015; Gómez-Armesto et al., 2015). Zn is linked with enzymes and participates in three plant functions: catalytic, coercive, and structural (Bravo et al., 2015). Ba is the trace element found at the highest concentrations in soil (Bravo et al., 2015), while Sb, As and Hg are non-essential trace elements. All these elements are named by Hooda, (2010) as PTEs, whose presence in soil poses a serious soil quality problem and a human health risk (Rascio and Navari-Izo, 2011; Sun et al., 2018).

The concentration of PTEs in plants depends on several factors, such as abundance and speciation ((bio)availability) in soil, type of plant and its age, depth of roots, among others (Cunha et al., 2014). The ability of plants to take up nutrients can be measured by the bioaccumulation coefficient (BAC), which is calculated as the ratios between the concentration of the element in a plant (any plant tissue, e.g., root, leaf, or fruit) and its content in soil (Kabata-Pendias, 2011; Cunha et al., 2014; Bravo et al., 2017), to observe the element’s bioavailability in soil (Bravo et al., 2017). The bioaccumulation coefficient (BAC) applied to PTEs describes the transfer from soil to plants, while the bioconcentration coefficient (BC) describes a plant’s ability to adsorb PTEs from soil when they appear in an available form (Gruszecka-Kosowska, 2020).

Many plants are used for direct human consumption as they form part of the population’s diet, such as fruit and cereals. The human health risk posed by eating them as part of their daily diet can be assessed and determined by calculating the non-carcinogenic and carcinogenic risks of the PTEs they contain (Gruszecka-Kosowska, 2019, 2020). Eating plants can also affect human beings indirectly via ruminant animals because they form part of the food chain and can also affect ruminant animals’ health (Aquilina et al., 2016; Pareja-Carrera et al., 2021).

According to the World Population Prospects, each state should promote its own research in relation to their agricultural regions and agroecosystems (UN, 2015). The National Secretary of Science and Technology (SENACYT) and the Institute for the Training and Use of Human Resources (IFARHU) of Panama promote a project in the abandoned Remance gold mine, where tailings are exposed to the climate conditions of wind and rain, which can affect surrounding soils and plants. The peasants who live within the old mine perimeter grow products for their own consumption and graze livestock, even in those areas very close to tailings. The objective of this study was to analyse the degree that mining activity affected flora in relation to the concentration of PTEs and their bioavailability by bearing in mind the human risk assessment and evaluating the health risk.

## Materials and methods

### Study area

The Remance gold mine is located in the village of Remance, a district of San Francisco, in the Veraguas province in the Republic of Panama, Central America. From a geological point of view, a hydrothermal alteration covers an area of some 10 km^2^, and the epithermal gold deposit is hosted on a bed of pyroclastic rocks (Nelson & Ganoza, 1999). The gold deposit comprises a system of veins in which the principal vein contains the largest ore quantities, along with minor, but still relevant, veins like Santa Rosa and Consuelo, which are subterranean and have sporadic outcrops (Nelson & Ganoza, 1999).

The mine has been exploited intermittently by different companies for over 200 years, between 1800 and 1998. The last exploitation company was “Minera Remance S.A”, which operated the mine between 1989 and 1999 (Nelson & Ganoza, 1999) by applying the cyanidation process to extract precious metal (Gómez, 2008). Nowadays the mine is abandoned, and there are still three tailing ponds with mining waste exposed to environmental conditions, which could be sources of pollution for soils, water bodies, and flora (González-Valoys et al., 2021a).

According to the Köppen climate classification map, the climate in the study area corresponds to the Ami type. It is a humid tropical climate, with the influence of monsoons, and an annual rainfall of > 2,250 mm that concentrates (60%) in the four wettest months (August-November). The rain rates of dry months (January-March) drop below 60 mm, and the average temperature of the coolest month is > 18 °C (Dirección de Meteorología de ETESA, 2007).

Pasture predominates in the old mine area, with stubble and shrubby vegetation no higher than 5 m and a few small mixed broadleaf forest patches. Some small settlements are found in the area, and the commonest annual crops are rice, sugarcane, and corn (Ministerio de Ambiente Panamá, 2012), as well as other crops like cassava, banana, beans, among others. Cattle raising and horse grazing are also observed.

### Sampling

**Fig. 1:**
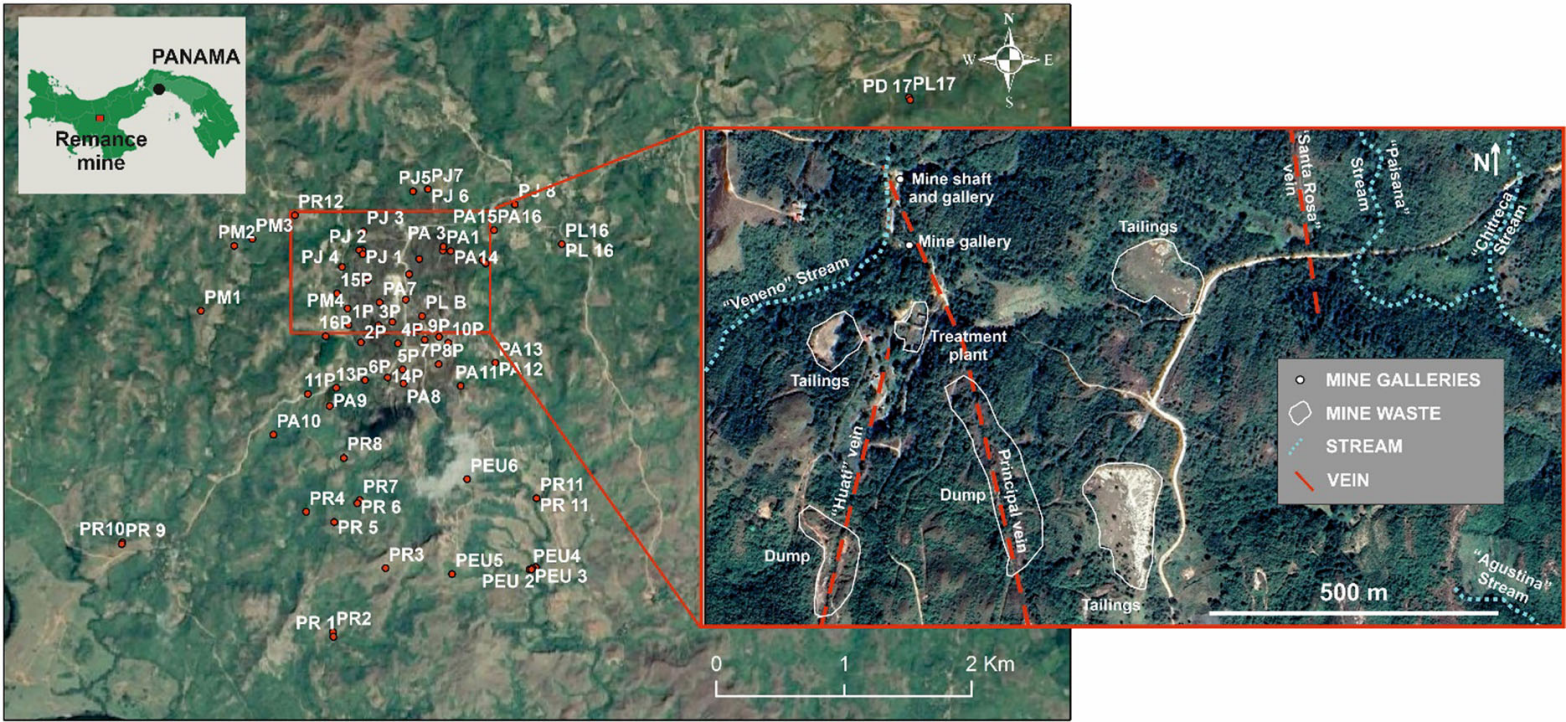
Location map of the plant samples taken within the Remance gold mine perimeter

Plant sampling was performed between May and June 2019, and in January 2020. Table 1 offers the collected samples, together with their common name, family, taxa and frequency for 75 samples, Table ST1 presents the coordinates. The location map of the samples appears in Fig. 1. The studied tissue was either leaves or edible plant parts. Together with each plant, a soil sample was collected to determine the BAC to evaluate the transfer of PTEs from soil to plants, and the available fraction was noted to evaluate the BC.

**Table 1:** plant samples taken for the study. Edible plants in bold. The common names in italics are in Spanish

Family	Taxon	Common name	Frequency
*Anacardiaceae*	*Anacardium excelsum (Bertero & Balb. ex Kunth) Skeels*	*Espavé*	3
*Annonaceae*	*Xylopia frutescens* Aubl	*Malagueto macho*	4
*Araceae*	morphospecies	–	1
*Araliaceae*	*Schefflera morototoni (Aubl.) Maguire, Steyerm. & Frodin*	*Mangabe*	1
*Asteraceae*	*Baccharis trinervis* Pers	–	1
*Asteraceae*	***Ayapana stenolepis (*Steetz) R.M. King & H. Rob**	***Tea leaves***	1
*Bombacaceae*	*Pseudobombax septenatum* (Jacq.) Dugand	*Barrigón*	2
*Boraginaceae*	*Heliotropium indicum L*	Turnsole, indian heliotrope	2
*Burseraceae*	*Bursera simaruba* (L.) Sarg	*Indio desnudo*	2
*Clusiaceae*	*Garcinia madruno (Kunth) Hammel*	*Satro*	1
*Connaraceae*	*Cnestidium rufescens* Planch	–	1
*Convolvulaceae*	*Ipomoea batatas* (L.) Lam	Yam or sweet potato	1
*Cyperaceae*	*Rhynchospora cephalotes* (L.) Vahl	Grass	2
*Dennstaedtiaceae*	*Pteridium caudatum (L.) Maxon*	Fern	2
*Dilleniaceae*	*Curatella americana* L	*Chumico*	4
*Euphorbiaceae*	*Mabea occidentalis* Benth	*Caciquillo*	2
*Euphorbiaceae*	***Manihot esculenta* Crantz**	**Cassava, *yuca***	1
*Fabaceae-Mimosoideae*	*Acacia mangium* Willd	*Acacia*	1
*Fabaceae-Mimosoideae*	*Acacia sp.*	*Acacia*	1
*Fabaceae-Mimosoideae*	*Calliandra magdalenae (Bertero ex DC.) Benth*	–	1
*Fabaceae-Mimosoideae*	*Cojoba rufescens* (Benth.) Britton & Rose	*Coralillo*	1
*Fabaceae-Mimosoideae*	*Zygia longifolia (Humb. & Bonpl. ex Willd.) Britton & Rose*	*Pichindé*	1
*Fabaceae-Papilionoideae*	*Andira inermis* (W. Wright) Kunth ex DC	*Harino*	4
*Gleicheniaceae*	*Dicranopteris pectinata* (Willd.) Underw	Fern	1
*Lauraceae*	*Nectandra* sp.	*Sigua*	1
*Lygodiaceae*	*Lygodium venustum* Sw	*Crespillo*	2
*Malpighiaceae*	*Byrsonima crassifolia* (L.) Kunth	*Nance*	2
*Malvaceae*	*Guazuma ulmifolia* Lam	*Guácimo*	3
*Malvaceae*	mophospecies	–	3
*Melastomataceae*	*Miconia argentea* (Sw.) DC	*Papelillo*	1
*Melastomataceae*	*Mouriri myrtilloides* (Sw.) Poir		1
*Moraceae*	*Brosimum alicastrum Sw*	*Berba, cacique,* breadnut	1
*Myrtaceae*	*Eugenia* sp.	*Guayabillo*	1
*Piperaceae*	*Piper leptocladum* C. DC	*Cordoncillo*	1
*Poaceae*	morphospecies	Grass*, pasto*	12
*Poaceae*	***Oryza sativa* L**	**Rice, *arroz***	1
*Poaceae*	***Zea mays* L**	**Corn, *maíz***	1
*Rubiaceae*	*Alibertia edulis* (Rich.) A. Rich	Trumpet	1
*Rubiaceae*	***Declieuxia fruticosa* (Willd.) Kuntze**	***Tea leaves***	1
*Rubiaceae*	*Genipa americana* L	*Jagua*	1
*Sapindaceae*	*Cupania americana L*	*Gorgojo,* weevil	1

The edible part was taken from edible plants, while 30–40 leaves were collected from the rest of the plant as composite samples using gloves and scissors. Samples were placed in a paper envelope and stored at room temperature before being analysed. Soil samples were collected at 0–30 cm deep inside a PVC tube, which was placed inside soil to obtain samples (González-Valoys et al., 2021b). Soil samples (approx. 3 kg each) were placed in a plastic bag using a plastic shovel to be stored at ambient temperature.

### Processing and analysing samples

Leaf samples were washed with deionised water to eliminate dust impurities, were left at ambient temperature for 4 days and then left to dry for 3 more days at 36 °C in a stove. Finally, samples were crushed by a domestic grinder to increase homogeneity. In the same way, soil samples were dried at ambient temperature, disaggregated with the help of a manual roller and sieved to less than 2 mm. The aliquots taken for the analysis (50 g) were further ground in an agate mortar until the diameter of the material was below 100 μm.

The elements Cu, Zn, As, Ba, Sb, and T-Hg were studied because in a previous study of the tailings from the abandoned gold mine, they are the PTEs that were above the value of the Panama soil standard (González-Valoys et al., 2021a). The Cu, Zn, As, Sb, and Ba determinations were made in both sample kinds, namely plants pressed into tablets and soil in a powder form**,** by energy dispersion X-ray fluorescence spectroscopy (ED-XRF) in Epsilon One equipment (PANalytical brand). Total Hg (T-Hg) was determined by Zeeman atomic absorption spectroscopy with high-frequency modulation of light polarisation (ZAAS-HFM) using commercial equipment Lumex RA-915 M with a pyrolytic attachment (PYRO-915 +). Certified reference materials were used to check both precision and accuracy: NIST 2710A (Montana soil) and LGC7162 (strawberry leaves). Recovery percentages between 80 and 100% (ED-XRF) and 95–100% (ZAAS-HF) were obtained.

Based on high As and Cu concentrations determined by the ED-FRX analysis, a set of 12 soil samples was selected to evaluate the BC. In this way, the sequential extraction in three stages proposed by the European Community Bureau of Reference (BCR) was carried out in accordance with the procedure described by Sahuquillo et al., (1999): in step 1 (S1) the exchangeable and bound to carbonates fraction is extracted with acetic acid; in step 2 (S2) the reducible fraction (bound to Fe and Mn oxides), is extracted with hydroxylamine hydrochloride; and in step 3 (S3) the oxidizable fraction (bound to organic matter and sulphides) is extracted using a digestion with hydrogen peroxide first and then ammonium acetate. This method is widely used for evaluating the fractionation of metals and has been applied to study a wide variety of solid samples, including different mining waste types (Marguí et al., 2006; García-Ordiales et al., 2019). Hence, the first three fractions were considered potentially labile or reactive fraction plant uptake or bioaccessible to humans (Kelepertzis & Stathopoulou, 2013; Madrid et al., 2007).

Measurements of Cu and As in the BCR extracts were taken by high-resolution atomic absorption spectroscopy (HR-AAS) in ContrAA-800 equipment (Analytik Jena brand) using the flame and the graphite furnace techniques, respectively. Samples were also subjected to microwave-assisted acid digestion with aqua regia according to EPA method 3051A (USEPA, 2007) to analyse pseudo-total concentrations (Higueras et al., 2017; Melaku et al., 2005). In all cases, solutions were filtered with Whatman filters (8 µm). As a quality control of total contents, analyses of blanks and random duplicates were performed. Certified reference material NIST 2710A was also digested and analysed in triplicate, with 95% and 98% recovery for Cu and As, respectively. Blanks and certified reference material BCR 701 were also used in the BCR extractions with recoveries between 95 and 102% for Cu (As is not certificated in this reference material).

### Soil to plant transfer indices

Two indices were used to determine the transfer of PTEs from soil to plant: BAC and BC. The BAC is a key component for quantifying differences in metal bioavailability by describing the transfer of PTEs from soil to plant (Gruszecka-Kosowska, 2019; Inacio et al., 2014). The ratios between the concentration of the element in the plant and the element concentration in soil was calculated (Bravo et al., 2015; Kabata-Pendias, 2011).

$${\text{BAC }} = {\text{ C}}_{{{\text{leaves}}\;{\text{or}}\;{\text{edible}}\;{\text{part}}\;{\text{plant}}}} /{\text{C}}_{{{\text{soil}}}}$$

where C _leaves or edible part plant_ is the concentration of a particular PTE (mg kg^−1^) in the leaves or edible part of the plant, and C _soil_ is the total concentration of a particular PTE in soil samples (mg kg^−1^).

*Bioconcentration coefficient (BC)*: describes the plant’s capacity to adsorb PTEs from soil when PTEs appear in an available form (Gruszecka-Kosowska, 2019; Inacio et al., 2014). BC is calculated as the ratios between the PTE concentration in leaves or edible parts and the available concentrations of PTE in soil (Wang et al., 2006):

$${\text{BC }} = {\text{ C}}_{{{\text{leaves}}\;{\text{or}}\;{\text{edible}}\;{\text{part}}\;{\text{plant}}}} /{\text{C}}_{{{\text{soil}}\;{\text{available}}}}$$

where C _leaves or edible part plant_ is the concentration of a particular PTE (mg kg^−1^) in the leaves or the edible part plant and C _soil available_ is the concentration of a particular PTE in soil samples (mg kg^−1^) obtained from the BCR three-stage sequential extraction procedure because it is considered potentially labile or the reactive fraction plant uptake (Kelepertzis & Stathopoulou, 2013; Madrid et al., 2007).

### Human health risk assessment

This assessment was performed by the following parameters: daily intake rate (DIR), average daily dose (ADD), hazard quotient (HQ), and carcinogenic risk (CR).

Daily intake rate (DIR) was calculated as the sum of consumed food (Gruszecka-Kosowska, 2019; WHO, 2005) which, in this case, included rice (grain), corn (grain), cassava (tuber), and tea leaves.

$${\text{DIR }} = \, \Sigma \, \left( {{\text{C}}_{{{\text{food}}}} \times {\text{ IR}}_{{{\text{food}}}} /{\text{BW}}} \right)$$

where C_food_ is the concentration of a particular PTE in food (rice, corn, cassava, tea leaves) (mg kg^−1^), IR is the ingestion rate (g person^−1^ day^−1^) in food and BW is body weight (70 kg for adults) (USEPA, 2011). Table 2 presents the IR values used to calculate the DIR for an adult and corresponds to: the IR value of Panama as reported in a consultancy by the FAO (Kennedy et al., 2021) for rice; the minimum value for America (García-Casal et al., 2018) for corn considering that Panama consumes corn-based products to a lesser extent than the rest of Central America; the values reported in Nigeria (Afolami et al., 2020) for cassava; an average value reported for Pakistan (commercial black tea brands) (Idrees et al., 2020), and China (tea leaves)(Zhang et al., 2018). Here “teas” are taken to correspond to the herbs used locally for infusions (*Ayapana stenolepis* and *Declieuxia fruticosa*).

**Table 2:** The IR values for different types of edible plants

Type of plant	IR (g person^−1^ day^−1^)	Reference
Rice, grain	125.2	Kennedy et al., (2021)
Corn, grain	50.0	García-Casal et al., (2018)
Cassava, tuber	42.0	Afolami et al., (2020)
Tea leaves	10.9	Idrees et al., (2020)/ Zhang et al., (2018)

The ADD was calculated as the sum of the consumed food (Gruszecka-Kosowska, 2019; USEPA, 1989):

$${\text{ADD }} = \, \Sigma \, \left( {{\text{C}}_{{{\text{food}}}} \times {\text{ IR}}_{{{\text{food}}}} \times {\text{ EF }} \times {\text{ ED }} \times { 1}0^{{ - {3}}} } \right) \, /{\text{ AT }} \times {\text{ BW}}$$

where C_food_ is the PTE concentration in the investigated food (mg kg^−1^), IR_food_ is the intake rate of cereals (g person^−1^ day^−1^), EF is exposure frequency: 365 d y^−1^, ED is exposure duration with 30 y for adults (USEPA, 2011), AT is the average time in days with ED × 365 for non-carcinogens, and 70 y × 365 for carcinogens (Gruszecka-Kosowska, 2019; USEPA, 2001), BW is body weight (70 kg) and 10^−3^ is a unit conversion factor.

The non-carcinogenic risk represents the risk of daily exposure to PTEs (Gruszecka-Kosowska, 2019). The HQ is the non CR, where a value of 1 refers to the threshold reference value as suggested by the US Environmental Protection Agency (Pan et al., 2019), and is calculated as follows (USEPA, 1989):

$${\text{HQ }} = {\text{ ADD}}/{\text{RfD}}$$

where HQ is the hazard quotient and RfD is the reference dose for a particular PTE. The RfD values for the PTEs (USEPA, 2019) in this study are presented in Table 3. The total non CR (HQt) value for the investigated PTEs was calculated as so (USEPA, 1989)

$${\text{HQt }} = {\text{ HQ}}_{{1}} + {\text{ HQ}}_{{2}} + \, \cdots \, + {\text{ HQ}}_{{\text{n}}}$$

where HQ are the hazard quotient values for the 1-n PTEs herein investigated.

**Table 3:** The RfD value of the non-carcinogenic elements and the SF for carcinogenic elements

Element	RfD (mg kg^−1^d^−1^)	SF (mg kg^−1^d^−1^)
Cu	4.0 × 10^–2^	1.7
Zn	3.0 × 10^–1^	
As	3.0 × 10^–4^	1.5
Sb	4.0 × 10^–4^	
Ba	2.0 × 10^–1^	
Hg	3.0 × 10^–4^	

The CR values of the PTEs from dietary exposure were calculated by the formula (Gruszecka-Kosowska, 2019; USEPA, 1989):

$${\text{CR }} = {\text{ ADD }} \times {\text{ SF}}$$

where CR is carcinogenic risk and SF is the oral slope factor over a lifetime for a particular PTE. The SF plays a key role being that the daily toxin intake results in an incremental risk of an individual developing cancer (Pan et al., 2019). Table 3 presents the SF values for the carcinogenic elements (Pan et al., 2019) in this study. The total CR value appears as the sum of the partial CR values (USEPA, 1989).

$${\text{CRt }} = {\text{ CR}}_{{1}} + {\text{ CR}}_{{2}} + \cdots + {\text{ CR}}_{{\text{n}}}$$

where CR are the carcinogenic risk values for the 1-n PTEs herein investigated.

### Statistical analyses

Microsoft Excel spreadsheets were used to manage the results. Minitab 15 was employed to analyse the statistical parameters of the analytical results.

## Results

### PTEs and BAC

The synthetic statistical parameters for the group of samples are provided in Table 4 and SF1, all the obtained results for plants are expressed in ST2. The Cu concentrations in plant leaves varied between 4.3 and 57.3 mg kg^−1^, while the BAC values indicated that Cu absorption was a weak to strong absorption accumulation in plants. It was remarkable that *Xylopia frutescens Aubl* was the species with the highest accumulation. The Zn concentrations in plant leaves were between 5.7 and 273.1 mg kg^−1^, while the BAC values indicated that Zn absorption went from weak to strong absorption accumulation in plants. In this case, Araceae *morphospecies* was the plant taxon with the most accumulation. The As concentrations in plant leaves were between < 0.1 and 54.5 mg kg^−1^, while the BAC values indicated very weak to strong absorption accumulation. The taxon with the most accumulation was *Poaceae morphospecies*. The Sb concentrations in plant leaves were between < 1.0 and 9.7 mg kg^−1^, while the BAC values denoted a weak absorption to strong accumulation, with *Declieuxia fruticosa* (Willd) Kuntze being the species with the most accumulation. The Ba concentrations in plant leaves went from < 5.0 to 319.9 mg kg^−1^, and BACs indicated very weak to moderate absorption. The Hg concentrations in leaves were between < 0.1 and 191.2 ng g^−1^, while the BAC values indicated a very weak absorption to strong accumulation, with *Anacardium excelsum (Bertero & Balb. ex Kunth) Skeels* being the taxon with the highest accumulation rate for these elements. All the plants herein indicated with maximum concentrations corresponded to non-edible plants.

**Table 4:** Value of the PTEs in leaves and soils for Cu, Zn, As, Sb, and Ba expressed as mg kg^−1^, Hg in ng g^−1^ and BAC per element

Element	Range plant	Mean plant	Stand. dev. Plant	Range soil^*^	Mean soil^*^	Stand. dev. soil^*^	Range BAC	Mean BAC	Stand. dev. BAC	Description	Plant with most accumulation or absorption
Cu	4.3–57.3	16.9	9.6	5.4–396.9	70.3	61.1	0.02–2.89	0.46	0.47	Weak absorption to strong accumulation	*Xylopia frutescens* Aubl
Zn	5.7–273.1	31.1	34.9	12.0–166.1	54.4	27.5	0.06–5.32	0.7	0.75	Weak absorption to strong accumulation	*Araceae morphospecies*
As	< 0.1–54.5	2.4	9.4	< 0.8–714.5	110.6	171	< 0.001–1.50	0.06	0.21	Very weak absorption to strong accumulation	*Poaceae morphospecies*
Sb	< 1.0–9.7	3.5	1.8	< 0.6–41.8	16.1	6.3	0.01–7.83	0.48	1.15	Weak absorption to strong accumulation	*Declieuxia fruticosa* (Willd.) Kuntze
Ba	< 5.0–319.9	31.1	45	40.0–743.2	310.9	166.8	< 0.001–0.93	0.14	0.2	Very weak to moderate absorption	*Anacardium excelsum (Bertero & Balb. ex Kunth) Skeels*
Hg	< 0.1–191.2	18.5	29.5	< 5.0–6470.0	276.1	797.7	< 0.001–2.38	0.27	0.42	very weak absorption to strong accumulation	*Anacardium excelsum (Bertero & Balb. ex Kunth) Skeels*

*Values of the PTEs in soil taken from González-Valoys et al., (2021b)

Table 5 is a compendium of the Cu, Zn, As, Sb, Ba, and Cu concentrations in plants from different countries around the world in both uncontaminated and contaminated areas to compare these values to those obtained at the Remance gold mine for edible products like rice (grain), corn (grain), cassava (tuber), tea leaves (medicinal plants), grass (leaves), and plants in general (leaves).

**Table 5:** Comparative table of the uncontaminated and contaminated sites in several countries for rice, corn, cassava, tea leaves, grass, and plants in general; in relation to the concentration of potentially toxic elements (Cu, Zn, As, Sb, Ba, and Hg in mg kg^−1^)

Plant	Site	Cu	Zn	As	Sb	Ba	Hg	Reference
Rice, grains	Uncontaminated sites-different countries		18.0	< 0.1				Kabata-Pendias, 2011
	Agricultural soils-China	2.7	18.0				0.004	Rothenberg et al., 2011
	Agricultural soils-Italy	4.8	24.6	< 0.1	1.1	9.3		Nadimi-Goki et al., 2014
	Agricultural soils-Sri Lanka	2.2	15.5	0.1				Rajatheja et al., 2021
	Contaminated site-different countries	4.0		1.2			4.900	Kabata-Pendias, 2011
	Remance mining area-Panama	5.2	17.9	0.2	4.4	12.1	< 0.001	This work
Corn, grains	Uncontaminated sites-different countries		30.5	1.8	< 2.0		0.037	Kabata-Pendias, 2011
	Agricultural soils-Brazil	1.7	17.5	< 0.1		2.7	< 0.030	Yada et al., 2020
	Agricultural soils-Poland	0.5	7.4	< 0.1	< 0.1		0.002	Gruszecka-Kosowska, 2020
	Agricultural soils-Czech Republic	1.4	6.5					Adaev et al, 2021
	Industrial area-Greece	2.3	16.0	0.2	0.4			Antoniadis et al., 2019
	Coal mining-contaminated soil-China	1.7	22.7			6.1		Hussain et al., 2019
	Contaminated site-different countries						0.105	Kabata-Pendias, 2011
	Remance mining area-Panama	4.3	22.0	0.2	4.7	11.7	< 0.001	This work
Cassava, tuber	Agricultural soils-Ghana		7.4					Danso et al., 2001
	Agricultural soils-Nigeria	11.2	< 0.1					Adejumo et al., 2019
	Remance mining area-Panama	7.5	9.0	< 0.1	4.1	18.5	< 0.001	This work
Tea, leaves	Uncontaminated sites-different countries	20.0					0.040	Kabata-Pendias, 2011
	Black tea-Pakistan	8.9	1.4					Idrees et al., 2020
	Remance mining area-Panama	19.2	88.8	0.6	4.7	35.5	0.002	This work
Grass, leaves	Uncontaminated sites-different countries	6.0	31.5	2.8				Kabata-Pendias, 2011
	Uncontaminated sites-Russia	14.6	47.4	< 0.1				Shtangeeva et al., 2020a, 2020b
	Uncontaminated sites-Russia	12.6	37.0	0.2	0.1	7.3		Shtangeeva et al., 2020a, 2020b
	Contaminated sites-different countries	42.0		31.2				Kabata-Pendias, 2011
	Coal mining-contaminated soil-China	18.5	86.4			41.4		Hussain et al., 2019
	Remance mining area-Panama	18.3	27.3	5.5	3.0	13.7	0.019	This work
Different types of plant leaves	Uncontaminated sites-different countries					7.5		Kabata-Pendias, 2011
	Uncontaminated sites-Russia	15.0	34.2	0.1				Shtangeeva et al., 2020a, 2020b
	Uncontaminated sites-Russia	9.2	50.0	0.2	0.1	19.0		Shtangeeva et al., 2020a, 2020b
	Coal mining-contaminated soil-China	7.1	43.1			25.4		Hussain et al., 2019
	Gold mining-Ethiopia	36.9	96.0	8.8	0.3			Getaneh & Alemayehu, 2006
	Remance mining area-Panama	16.9	31.1	3.4	3.9	36.5	0.021	This work

For rice, the average Cu concentration value at Remance (5.2 mg kg^−1^) was slightly higher than that reported by Kabata-Pendias (2011) for contaminated sites (4.0 mg kg^−1^), while the As contents (0.2 mg kg^−1^) were higher than the value for uncontaminated sites (0.005 mg kg^−1^) but lower than the reference level for contaminated sites (1.2 mg kg^−1^) (Kabata-Pendias, 2011). The concentrations of Sb (4.4 mg kg^−1^) and Ba (12.1 mg kg^−1^) were higher than those reported in agricultural soils in Italy (1.1 mg kg^−1^, 9.3 mg kg^−1^, respectively) (Nadimi-Goki et al., 2014). The average value of the Zn concentrations (17.9 mg kg^−1^) was similar to the values reported for uncontaminated sites (18.0 mg kg^−1^) (Kabata-Pendias, 2011) and in agricultural areas (15.5–24.6 mg kg^−1^) (Nadimi-Goki et al., 2014; Rajatheja et al., 2021; Rothenberg et al., 2011). Finally, the Hg concentrations were lower than the detection limit (< 0.001 mg kg^−1^).

For corn, Cu, Zn, As, Sb, and Ba were higher than in agricultural soils (Adaev et al., 2021; Gruszecka-Kosowska, 2020), while Zn was higher than cassava in agricultural soils (Danso et al., 2001). In tea leaves, the average Cu concentration fell within the same range as in uncontaminated areas (Kabata-Pendias, 2011), and Zn concentrations were much higher than those reported for commercial tea by Idrees et al (2020). In grass and plants, Cu, As, Sb, and Ba were higher than in the uncontaminated sites reported by Shtangeeva et al., (2020a, b).

### Statistical analysis

**Fig. 2:**
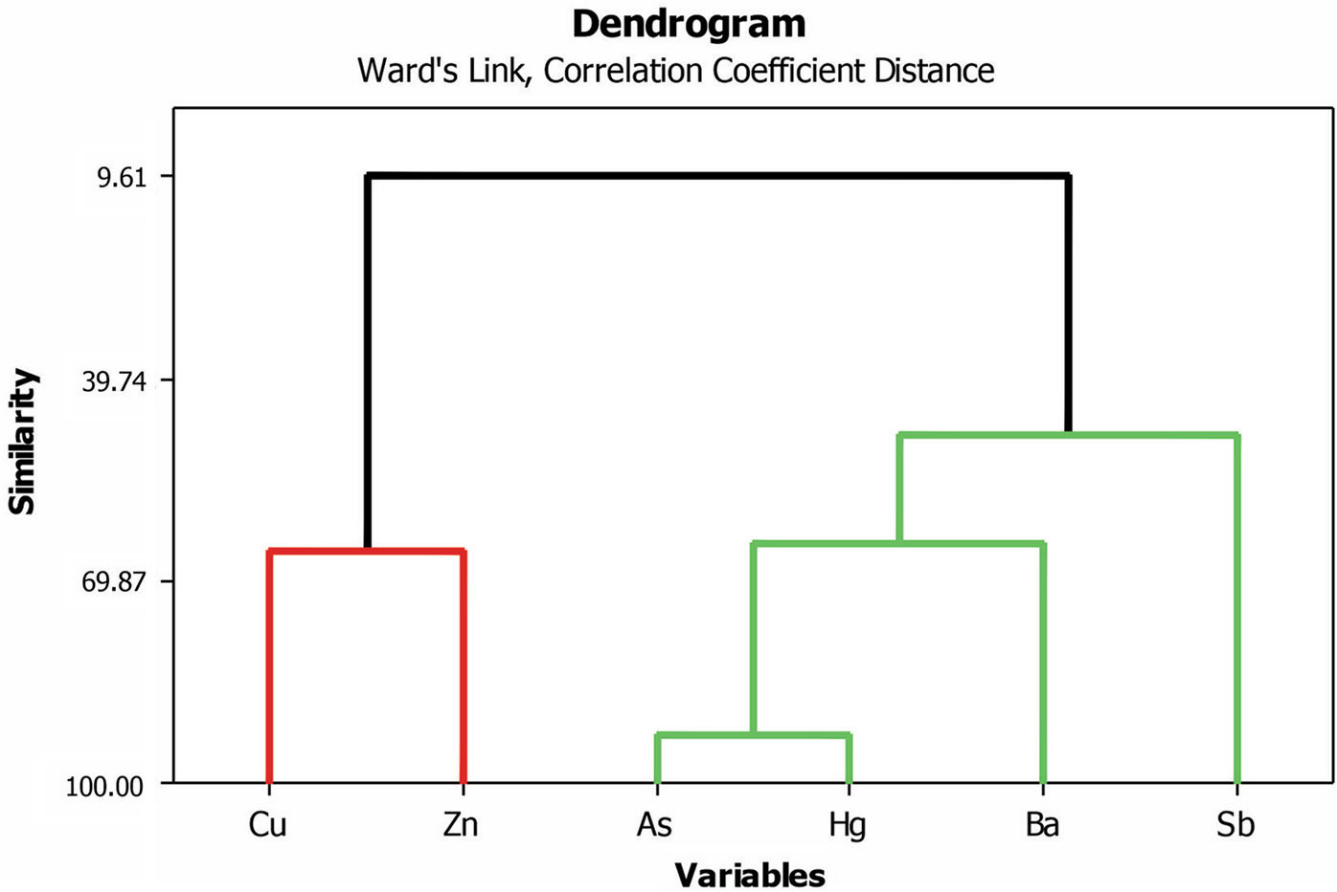
Dendrogram shows results of cluster analysis (Ward's method) and linkage distance between parameters of the PTEs found in the leaves samples

Figure 2 presents the dendrogram for the PTEs studied in Remance plant leaves and ST3 presents Pearson’s correlation. Pearson’s correlation showed that Cu was significantly related to Zn, meanwhile, As was related both to Ba and Hg, and Ba appears to be related to Hg. After a multivariate analysis, the relation among these six PTEs is displayed in the dendrogram of Fig. 2. This statistical approach clearly separated PTEs into two subgroups: one with Cu and Zn, and another including As, Hg, Ba, and Sb.

### Transfer of PTEs from soils to plants

**Fig. 3:**
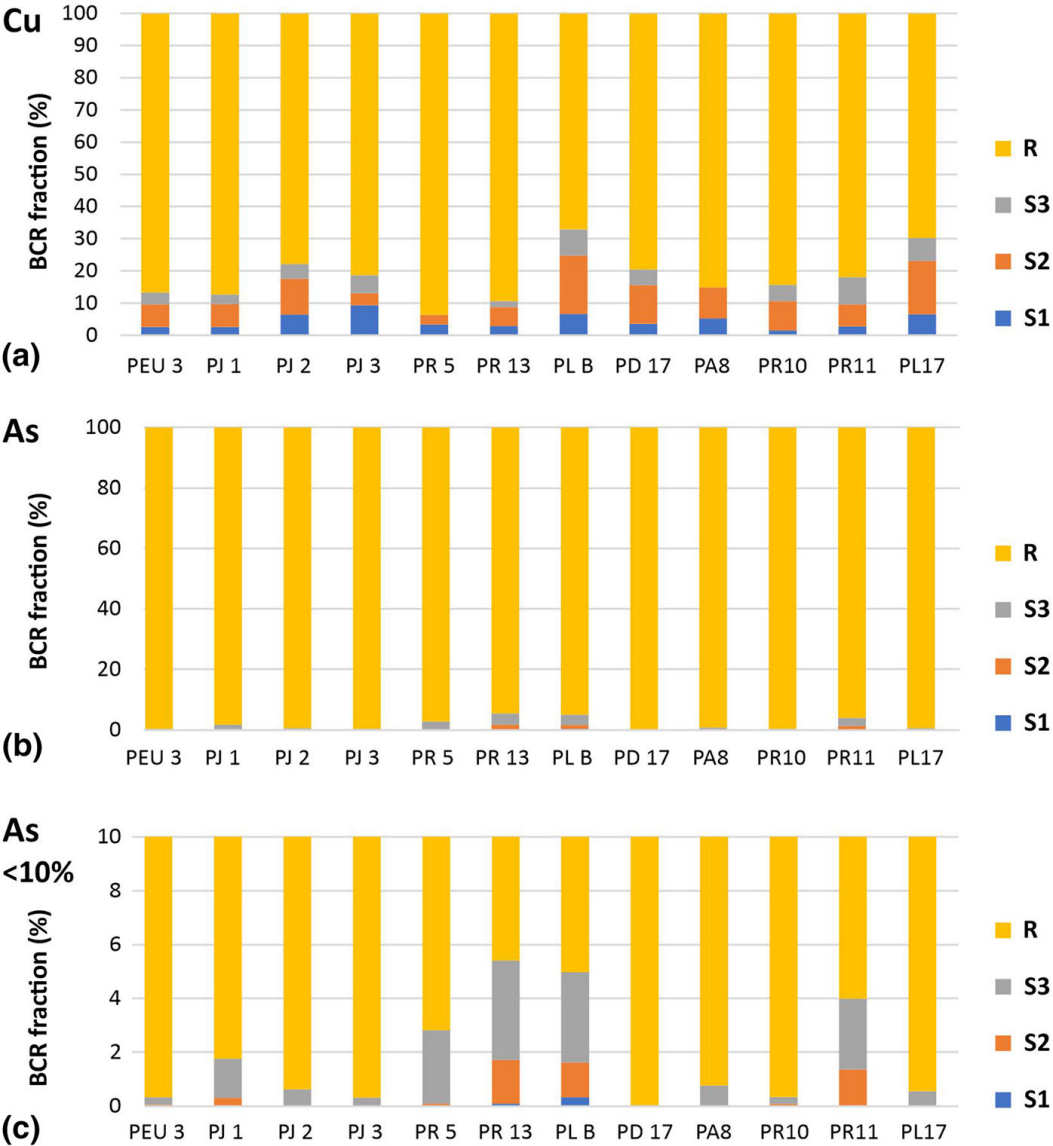
Combined bar graph for the BCR fractions and the residual in the plant-associated soil samples. **a** Cu fraction. **b** As fraction. **c** Detail of the fraction of As less than 10%

Figure 3 shows a combined graph of the percentages taken in each step of the BCR sequential extraction for Cu and As, respectively. The total extracted PTEs are displayed in ST3. It is possible to consider the first three BCR steps (S1 + S2 + S3) to be the fractions, including the potentially labile or reactive species, while the residual fraction can be taken as unavailable for transport, plant uptake, or as bioaccessible to humans (Madrid et al., 2007; Kelepertzis et al., 2013). The first fraction corresponds to the water-soluble fraction, which is easily exchangeable and interpreted as the most mobile and bioavailable for the environment (Pérez-López et al., 2008). Fraction 2 (metals bound to oxides Fe and Mn) and fraction 3 (complexed with sulphides and organic matter) can be mobilised under increasing reducing or oxidising conditions, respectively (Kelepertzis et al., 2013). For Cu, fractions 1, 2 and 3 averaged 4.45, 9.15 and 4.34%, respectively, with an average total labile fraction of 17.94% and fraction 2 with the highest contribution (Fig. 3). For As, fractions 1, 2 and 3 averaged 0.04, 0.40 and 1.39%, respectively, with an average total labile fraction of 1.82% and fraction 3 with the highest contribution (Fig. 3).

**Fig. 4:**
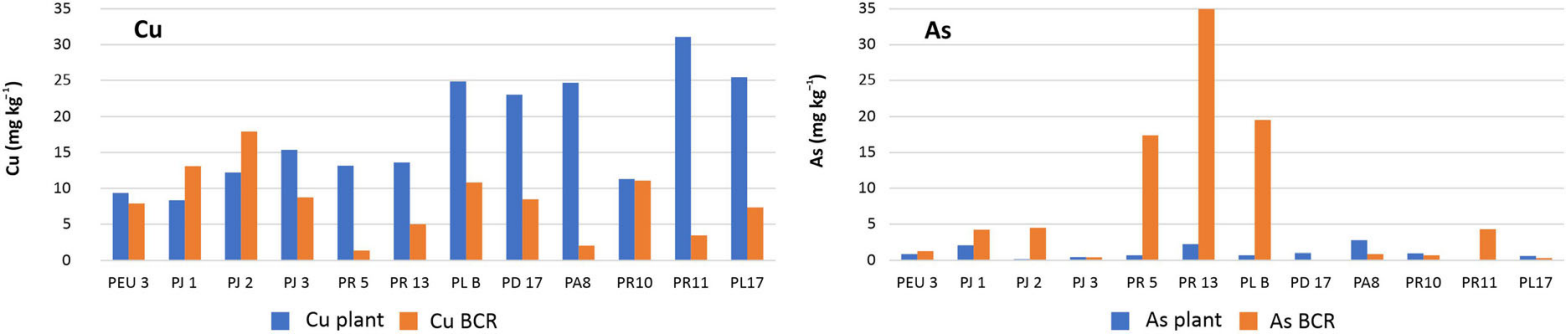
Bar graph comparing Cu and As concentration in leaves and available fraction (BCR S1 + S2 + S3)

Table ST4 presents the BAC and BC for a group of samples with high As and Cu contents in soil. These coefficients were used to evaluate the bioavailability of PTEs, and the plant’s capacity to bioaccumulate Cu and As and to bioconcentrate their available fractions. Figure 4 shows a bar graph to compare the fraction available in soil (obtained by BCR) and the concentration in the leaves of plants for Cu and As. Cu is seen as an essential element for plants and appears as being more available in soil (BCR), while plants show good uptake capacity and often high accumulation rates (average BC 3.97). As, which is scarcely available in most soils (mean BC: 0.88), also has lower uptake rates.

**Fig. 5:**
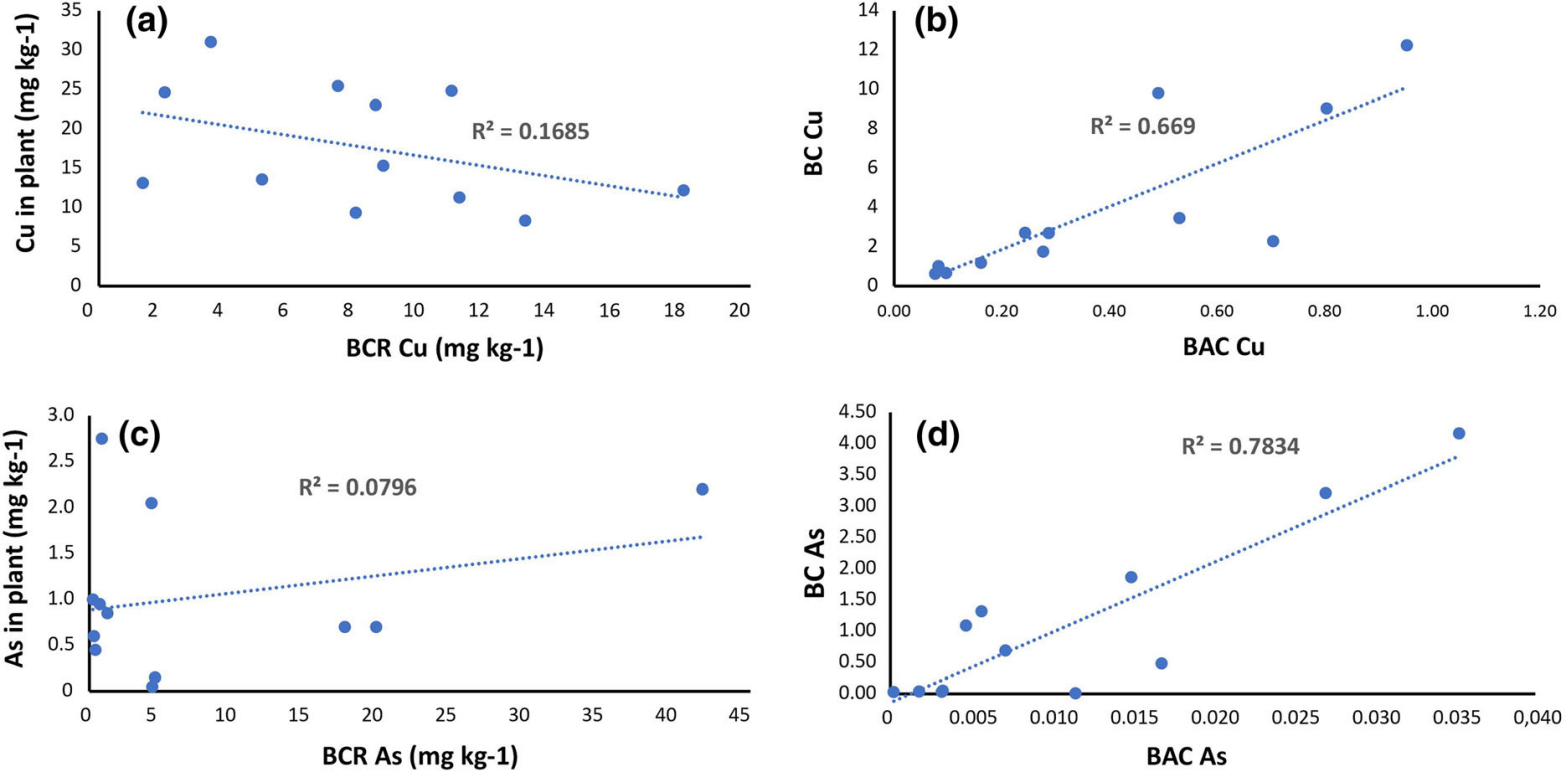
**a** Cu concentration in the plant vs available Cu concentration in soil (BCR). **b** Bioconcentration and bioaccumulation of Cu in the plant. **c** As concentration in the plant vs available As concentration in soil (BCR). **d** Bioconcentration and bioaccumulation of As in the plant

Figure 5a shows the correlation detected by Pearson’s test between the Cu concentration in plants and the Cu fraction available in soil, which is weakly negative. Figure 5c shows the relation between the As concentration in plants and the As fraction available in soil. No clear correlation is noted, albeit a very weakly positive one, which seems to be dominated by having low As absorption concentrations available in soil. Figures 5b and d show the positive and closer relationship between the BAC and BC indices for Cu and As, respectively.

### Human health risk assessment

#### Daily intake rates

Table 6 shows the DIR values of PTEs for the edible products obtained from the Remance gold mine. The inhabitants’ diet is based on products like rice, corn, or cassava, which are produced locally and consumed daily, with tea leaves consumed sporadically as medicinal tea. The DIR values of each edible product are compared to the provisional maximum tolerable daily intakes (PMTDI) (mg kg^−1^ bw day^−1^) (Gruszecka-Kosowska, 2020) as so: Cu 0.5 (FAO/WHO, 2001), Zn 1 (FAO/WHO, 2001), As 0.0021 (FAO/WHO, 1989), Sb 0.006 (WHO, 2008), Ba 0.02 (EU, 2012), Hg 0.0006 (FAO/WHO, 2011). The values of Cu DIR (2.024 to 9.301), Zn DIR (5.370 to 32.015), Sb DIR (0.716 to 7.780), and Ba DIR (4.562 to 21.642) exceeds the PMTDI in all foods, while As DIR (0.078 to 0.268) exceeds in food, except for cassava, and the Hg DIR is only marked in tea leaves (0.0008 to 0.0028) and exceeds the PTMDI.

**Table 6:** The DIR (mg kg^−1^ day^−1^), values of PTEs for the food products obtained from the Remance gold mine

Edible plants	ID	DIR Cu	DIR Zn	DIR As	DIR Sb	DIR Ba	DIR Hg
Rice, grain	PR15	9.301	32.015	0.268	7.780	21.64	0.00009*
Corn, grain	PR16	3.036	15.679	0.107	3.321	8.36	0.00004*
Cassava, tuber	PR8	4.470	5.370	0.030*	2.430	11.10	0.00003*
Tea leaves	PM1	2.024	8.074	0.101	0.732	4.56	0.00078
Tea leaves	PM4	3.963	19.550	0.078	0.716	6.49	0.00280
PMTDI		0.500	1.000	0.002	0.006	0.02	0.00060

*PMTDI*: provisional maximum tolerable daily intakes (mg kg^−1^ day^−1^)

*Calculations use the half of the detection limit

#### The non-carcinogenic risk of PTEs

**Fig. 6:**
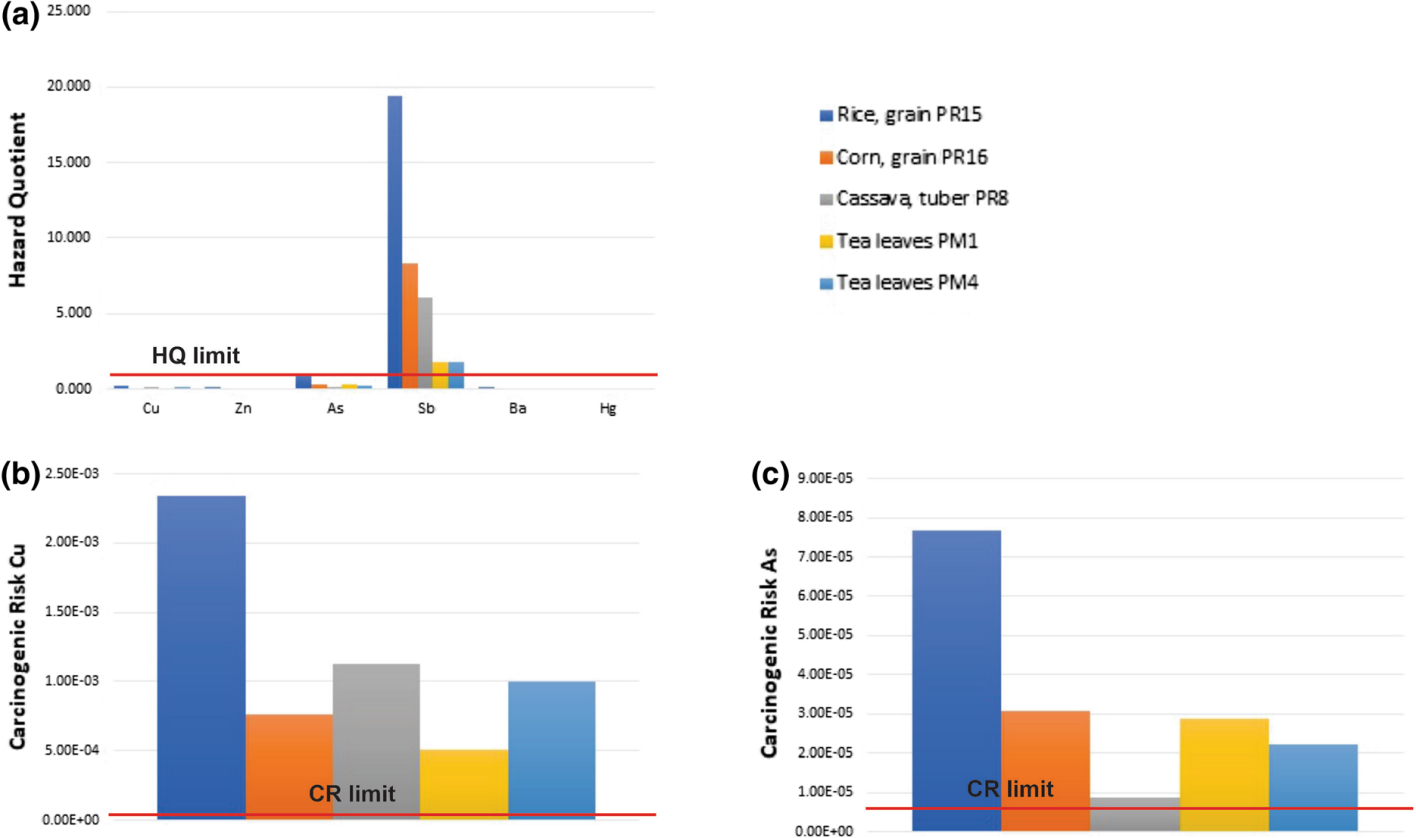
**a** Bar chart for non-carcinogenic (HQ) risk for PTEs in edible plants from the Remance gold mine. **b** Carcinogenic risk for Cu in edible plants studied for the Remance gold mine. **c** Carcinogenic risk for As in edible plants studied for the Remance gold mine

The non CR of PTEs was evaluated with the HQ, which was set at 1 (USEPA, 1989). Values exceeding 1 were considered a non CR. Figure 6a shows the HQ for the PTEs of the studied edible plant and ST5 values. As we can see, the HQ value was exceeded by Sb in them all and in this order: rice > corn > cassava > tea leaves (19.451 > 18.304 > 6.075 > 1.830). Cu, Zn, As, Ba, and Hg did not exceed the value of 1 for HQ. The total HQ value (sum of the HQ for PTEs) of all the edible plants exceeded 1, which means that it represents a non CR.

#### The carcinogenic risk of PTEs

The acceptable CR risk level was set to equal 1 × 10^–6^ for an individual PTE and to equal 1 × 10^–4^ for the sum of carcinogenic PTEs (USEPA, 1989). Values exceeding this are considered a CR. Figure 6b shows the CR for Cu and Fig. 6c for As and the ST5 includes the complete values. The acceptable CR value is exceeded by As in rice (7.67 × 10^–5^), corn (3.06 × 10^–5^) and tea leaves (2.22 × 10^–5^ to 2.89 × 10^–5^). Excess Cu was obtained in all the edible plants (5.10 × 10^–4^ to 2.34 × 10^–3^) in this order: rice > cassava > tea leaves > corn. This is the same order for the total CR.

#### Animal nutrition for ruminants

In the Remance mining area and its surroundings, peasants perform subsistence livestock work and graze horses. The mean Cu value in grass (*Poaceae morphospecies*) was 18.3 mg kg^−1^ and was 16.9 mg kg^−1^ in plants in general. These values exceed the maximum authorised for Cu (10 mg kg^−1^) for complete feed requirements in animal nutrition for ruminants (e.g., cattle, cows, and horses) of the National Research Council, USA (Aquilina et al., 2016; López-Alonso & Miranda, 2020; NRC, 2001). The mean Zn value in grass was 27.3 mg kg^−1^, and 31.1 mg kg^−1^ in plants in general. Both these values exceed the estimated value of the daily diet requirement for cattle for Zn (22.8 mg kg^−1^) (NRC, 2001). For As, Ba, Sb, and Hg, the National Research Council of the USA does not establish an estimated value for the daily diet requirements of cattle.

## Discussion

Given that the soils and plants in the surroundings of the abandoned Remance gold mine present high concentrations of PTEs, such as Cu, Zn, As, Sb, Ba, and Hg, associated with mineralisation (Nelson & Ganoza, 1999), it is essential to identify the degree to which plants, and especially the crops grown by farmers like rice, corn, cassava, among others, are affected (Ministerio de Ambiente Panamá, 2012). It is also necessary to identify the risks for livestock and as collateral risks for human health. The mean concentration of the PTEs in the leaves of a diversity of studied plants comes in this order, Zn = Ba > Cu > Sb > As > Hg, while BAC is related to the total amount of PTEs present in soil, and the degree to which a plant absorbs them comes in this order, Zn > Sb > Cu > Hg > Ba > As. All this indicates that essential trace elements like Zn and Cu (Arif et al., 2016) are absorbed by plants and accumulate more than non-essential elements (Bravo et al., 2015) like Hg, Ba, and As.

The exception can be Sb, which being non-essential, has been strongly absorbed and accumulated by plants (Mykolenko et al., 2018), even as As, which is in larger total concentrations in soil, evidencing the availability of these PTEs, which was corroborated with the BCR extraction (where the labile or available fractures are extracted for transport and plants) (Madrid et al., 2007; Kelepertzis et al., 2013) for As and Cu, where Cu was much more available than As.

For the soil–plant transfer of PTEs, a weakly negative linear regression between the Cu concentrations in plants versus the available Cu fraction in soil (BCR) was found. Although Cu is an essential element for plants, it is toxic for them if it appears in soil in large quantities (Adrees et al., 2015; Kumar et al., 2021; Rather et al., 2020; Shabbir et al., 2020). The relation between bioavailability indices BAC and BC (Kelepertzis et al., 2013) was positive, which reveals that plants’ ability to bioaccumulate Cu is enhanced, as does its ability to bioconcentrate it when Cu is available in soil.

The scenario is different for As because the relation between the elements in plants and in soil is not as clear. Although this relation was very weakly positive, it seemed to be dominated by having low absorbed As concentrations available in soil. This is a general rule, except for *Schefflera morototoni,* which absorbs As more efficiently by having mechanisms to tolerate and accumulate this toxic element. One remarkable fact is that, although As is more available in soil, plants do not always absorb it more, mainly because it is not an essential element (Ackova, 2018) and can be more related to each plant species’ capacity to exclude or tolerate this PTE (Chamba et al., 2017; Dixit et al., 2015). The relation between bioavailability indices BAC and BC was positive, which indicates that a plant’s ability to bioaccumulate As increases, as does its ability to bioconcentrate As when it is available in soil.

The mean concentration of both Cu and Zn in the leaves of the plants around the Remance gold mine, compared to plants from other parts of the world, fell within the ranges known for between uncontaminated (Shtangeeva et al., 2020a, 2020b) and contaminated zones (Hussain et al., 2019), while the As, Sb and Ba values were similar to those reported from contaminated areas (Getaneh & Alemayehu, 2006; Hussain et al., 2019). More specifically, the Cu concentrations in grass were similar to those from contaminated areas (Hussain et al., 2019), while As, Sb and Ba obtained higher values than those reported in uncontaminated areas (Shtangeeva et al., 2020a, 2020b), and the Zn concentrations were similar to those from uncontaminated areas (Kabata-Pendias, 2011). All these values imply harmful effects on the health of the cattle grazing in the study area for these PTEs because they are higher than those recommended for the animal nutrition of ruminants (Johnsen & Aaneby, 2019), as is the case of Cu and Zn (NRC, 2001). However, there are no estimated requirements set for cattle according to the National Research Council, USA, for the other PTEs (As, Sb, Ba, and Hg).

The human health risk posed by eating edible plants grown in areas with PTEs can be evaluated with the PMTDI (Gruszecka-Kosowska, 2020). This value was exceeded for Cu, Zn, Sb and Ba in all the studied edible plants (rice, corn, cassava, tea leaves), and for As in rice, corn and tea leaves, and for Hg only in tea leaves. Although some of these elements can be considered essential for plants or humans, they can be toxic to human health if consumed in excess, such as Cu, which brings about abnormalities in the nervous system, liver and kidneys, and even death, or Zn, which reduces the immune function and HDL cholesterol, and also causes fever. Non-essential PTEs can cause cirrhosis, cancer of the skin, liver and lungs, or embryo theratogenesis (As), respiratory system damage (Sb), gastroentheritis, muscle paralysis, ventricular fibrillation and extrasystoles (Ba), neurological damage (mercurialism), asthenic-vegetative syndrome or Minamata disease, kidney damage, toxicity to foetus and teratogenic embryo (Hg) (Bini & Wahsha, 2014).

The HQ values, with which the non-carcinogenic risk of edible plants is evaluated (Gruszecka-Kosowska, 2020), were exceeded for Sb, which places rice, corn, cassava, and tea leaves at risk levels. The long-term intake of small amounts of Sb may induce chronic antimony poisoning, while Sb exposure has been shown to induce DNA damage and oxidative stress, and to generate reactive oxygen species (ROS) causing apoptosis. As Sb geochemical behaviour is similar to that of As, it is likely that DNA damage induced by Sb follows similar pathways to those for As (Bini & Wahsha, 2014; Franco et al., 2009).

The acceptable CR was surpassed by all the edible plants for Cu, and also for As in rice, corn and tea leaves, which meant that the total acceptable CR was exceeded by all the studied edible plants and posed a risk for the health of the people who eat them in the studied mining area. One of the most important risks could come through As as long-term exposure can lead to skin lesions, internal cancers, neurological problems, pulmonary disease, peripheral vascular disease, hypertension and cardiovascular disease, and diabetes mellitus (Jaishankar et al., 2014; Smith et al., 2000).

The Remance gold mine is an abandoned mine. When abandoned mines are not properly shut down, they pose an environmental problem that also affects the inhabitants of their surroundings (Kaninga et al., 2020; Khlelifi et al., 2020). Therefore, environmental surveillance programmes need to be set up to avoid harming populations.

## Conclusion

The flora and crops of the Remance gold mine bioaccumulated the herein studied PTEs in this order: Zn > Sb > Cu > Hg > Ba > As. This finding indicates that this area has absorbed mostly essential elements like Zn and Cu along with Sb which is non-essential but has a very high affinity to be absorbed by plants. Of the major elements in soil, such as As and Cu, Cu was more available than As. This revealed that plants bioconcentrated Cu more than As despite As found in a larger total quantity in soil.

The BAC *vs.* BC relation was positive for both the tested Cu and As elements, which denotes that plants’ ability to bioaccumulate and bioconcentrate is linked with the availability of elements in soil.

The relationship between the Cu concentration in plants and the amount of Cu available in soil was weak and not very significant, as is the case for As. What this implies is that the amount of As available in soil was not directly linked with its concentration in plants, and this could, in turn, be linked with the mechanisms that each plant species possesses to absorb and bioaccumulate, or exclude, As.

The average Cu and Zn concentrations present in the grass and plants around the Remance gold mine exceeded the recommended requirements for the animal nutrition of ruminants according to the National Research Council, USA. So this could pose some health risks for the livestock grazing in this area.

Sb was the PTE that posed the main non CR. As and Cu were the PTEs that represented a CR because they exceeded the acceptable CR limit in the studied edible plants (rice, corn, cassava, tea leaves) that are planted and consumed by peasants as part of their daily diet.

We recommend the study area being bioremediated to reduce the posed risk for the environment and the people inhabiting the area.

## Supplementary Information

Below is the link to the electronic supplementary material.

Supplementary file1 (JPG 1185 kb)

Supplementary file2 (XLSX 39 kb)
