# Supplementary figures and images for: An approach for evaluating the bioavailability and risk assessment of potentially toxic elements using edible and inedible plants—the Remance (Panama) mining area as a model
Source: Environ Geochem Health. 2021 Oct 22;45(1):151–70. doi: 10.1007/s10653-021-01086-8 (PMC9867682; doi:10.1007/s10653-021-01086-8)

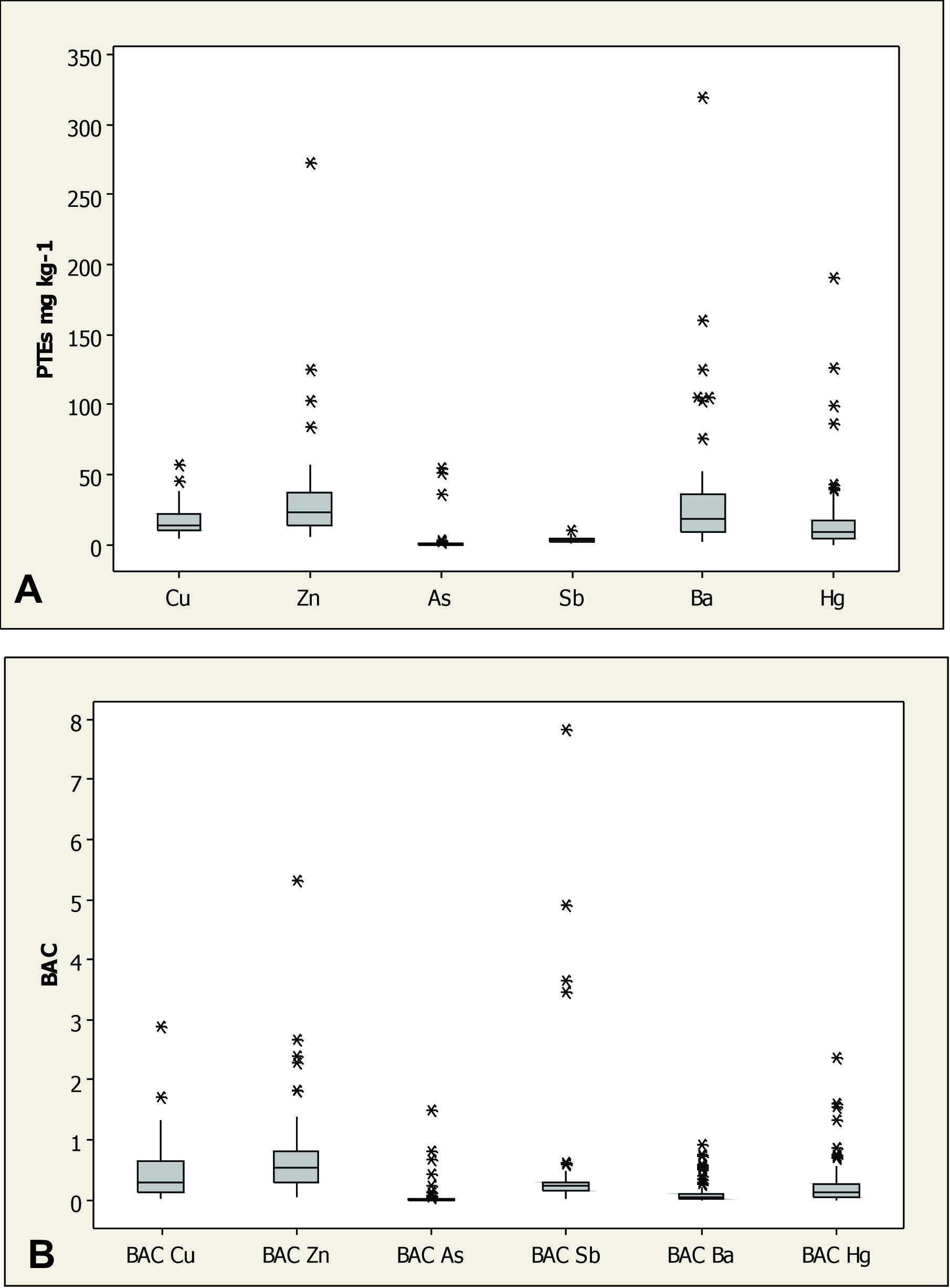

Supplement: Supplementary file 1 — Supplementary file1 (JPG 1185 kb) [file 10653_2021_1086_MOESM1_ESM.jpg]
